# Cardiovascular phenotyping of children and adolescents with post-COVID syndrome (PCS) at initial diagnosis - a prospective observational single-center study

**DOI:** 10.3389/fcvm.2026.1665734

**Published:** 2026-03-16

**Authors:** Hosan Hasan, Dagmar Hohmann, Klea Hysko, Sarah Ibahrine, Valentina Skeries, Katharina Dold, Gesa Helen Pöhler, Martin Wetzke, Georg Hansmann

**Affiliations:** 1 Department of Pediatric Cardiology and Critical Care, Hannover Medical School, Hannover, Germany; 2 European Pediatric Pulmonary Vascular Disease Network, Berlin, Germany; 3 Department of Pediatric Pulmonology, Allergology and Neonatology, Hannover Medical School, Hannover, Germany; 4 Department of Diagnostic and Interventional Radiology, Hannover Medical School, Hannover, Germany; 5 Division of Pediatric Cardiology, Pediatric Heart Center Vienna, Children’s Hospital, General Hospital Vienna (AKH), Medical University of Vienna, Vienna, Austria; 6 Department of Anesthesiology and Intensive Care Medicine, University of Tübingen, Tübingen, Germany

**Keywords:** cardiac MRI, echocardiography, pediatric cardiology, post-COVID syndrome, strain analysis

## Abstract

**Background:**

Post-COVID Syndrome (PCS) is an emerging, highly relevant topic in public health as it negatively affects quality of life and educational/work performance at all ages. To date, there is hardly any robust data on cardiac function in PCS (Long-COVID) available, particularly not in children and adolescents. The aim of this study was to conduct deep cardiovascular phenotyping in pediatric patients with PCS at initial presentation using cardiac MRI and echocardiography, including strain/tissue tracking analysis.

**Methods:**

Prospective, single center cohort study at Hannover Medical School, Germany (10207_BO_K_2022). PCS was defined as follows: persistent symptoms such as reduced physical performance, poor concentration, mood symptoms, headaches, sleep disorders and dysosmia, for at least 12 weeks after PCR-confirmed SARS-CoV-2 infection. A total of 100 pediatric patients (age 7–18 years, 56 female) and 20 age/gender matched healthy controls (age 8–18 years, 12 female) were enrolled between 03/2022 and 11/2023. Data collection consisted of 12-lead electrocardiogram (ECG), protocol-driven echocardiography (Echo; Philips EPIQ 7 ultrasound system, Philips Medical Systems), including tissue Doppler Echo and biventricular strain analysis (TOMTEC, Philips). 42 of the 100 PCS patients (age 9–18 years, 26 female) and 28 age/gender matched healthy controls (age 8–18 years, 14 female) received comprehensive cardiac magnetic resonance imaging (CMR; 3.0 T MRI system Magnetom Vida, Siemens Healthineers), including cine mass/volumes quantification in short axis (SAX) and tissue tracking (strain) analysis of the RV and LV (cvi 42). Laboratory studies included serum NTproBNP and Troponin c. Data are presented as mean ± SEM.

**Results:**

CMR-derived RV global radial strain (RVGRS) (22.6 ± 1.00% vs. 27.1 ± 1.13%; *p* = 0.003), and RV global circumferential strain (RVGCS) (−13.5 ± 0.55 vs. −15.2 ± 0.51%; *p* = 0.045) were significantly decreased in PCS vs. CON. Children with PCS also tended to have mildly reduced RVEF (50.9 ± 0.80 vs. 53.5 ± 0.66%; *p* = 0.259). RV mass index was increased in PCS compared to CON (19.06 ± 0.47 vs. 16.4 ± 0.53 g/m^2^; *p* = 0.0002), though in normal range referred to age-appropriate normal values. In contrast, CMR-derived LV variables (LVEF, LVEDV, LVESV, LV mass), including tissue tracking (strain) analysis (LVGLS, LVGCS, LVGRS), revealed similar values in PCS and control subjects. ECG and Echo data analysis did not show significant differences in PCS vs. CON.

**Conclusion:**

PCS is associated with decreased CMR-based radial and circumferential RV contractility (RVGRS, RVGCS) and slightly increased RV mass in children with PCS compared to healthy, age/gender matched controls. In contrast, LV contractility (strain) and mass were not affected. CMR feature tracking (strain) appears to be more sensitive than echocardiographic strain analysis. Whether the aforementioned RV alterations are causal for the reported cardiopulmonary exercise limitations in pediatric PCS is unknown, and should be investigated further.

## 1 Introduction

The COVID-19 pandemic has had a significant impact on the health of people of all ages worldwide, including children and adolescents. While most children show mild or asymptomatic courses of the disease, there is increasing evidence that they may also be affected by long-term health consequences, particularly with regard to cardiac function. Studies have shown that COVID-19 can affect not only the respiratory system but also the cardiovascular system, which can lead to a variety of cardiovascular complications (1, 2). Post Covid syndrome (PCS) is presently defined by the German Society for Pediatric Medicine (DGKJ) and the National Institute for Health and Care Excellence (NICE) as typical COVID symptoms that last at least 12 weeks after PCR confirmed SARS-CoV-2 infection and can not be explained by other diagnosis (3). Approximately 100 symptoms have now been described related to PCS, all of which can also occur in other diseases, meaning that the diagnosis of PCS remains a diagnosis of exclusion. PCS limits the quality of life of children, adolescents and their parents. In a large pediatric study (4) including children (7–16 years) with PCS, the most common symptoms were loss of smell/taste, fatigue, lack of concentration, respiratory symptoms, dizziness, chest pain and muscle weakness. Also mental health symptoms such as dysthymia seem to be prominent in children with PCS (5). Overall, PCS is a very complex and challenging clinical diagnosis, defined and treated differently in different centers and studies (4, 6). The actual cardiopulmonary risk of children suffering from PCS is still unclear (7). The aim of our study is the accurate cardiac phenotyping, including cardiac function and morphology, in children with PCS by using methods such as cardiac magnetic resonance imaging (CMR) (gold standard for the assessment of cardiac function), echocardiography (Echo) and electrocardiogram (ECG). An age-matched healthy control group is intended to objectively determine possible deviations to the healthy pediatric population. In particular, the detailed investigation of myocardial contractility in PCS patients using strain analysis, determined by CMR and Echo, should provide possible myocardial functional impairments that may indicate long-term inflammatory processes. Identification of an altered cardiac function and morphology may indicate possible pathophysiological mechanisms in the long-term course of Covid-19 disease and should be investigated further. The present work aims to emphasize the need for comprehensive cardiological evaluation and follow-up in children with PCS and to contribute to a better understanding of the long-term cardiovascular consequences of Covid-19 in children and adolescents.

## 2 Methods

### 2.1 Study population

This is a prospective observational single-center study including a total of 100 children (mean-age 13.2 ± 2.9 years; 56 female; mean-weight 52.9 ± 16.2 kg, mean-height 161.2 ± 15.8 cm, Table 1) with PCS, who presented in the pediatric PCS outpatient clinic of Hannover Medical School between March 2022 and July 2023. The study design included two separate age-matched healthy control groups, each serving a different purpose:

1. A control group of 20 children who underwent ECG and Echo examinations for comparison of these cardiac parameters.
2. A control group of 28 children who underwent comprehensive cardiac magnetic resonance imaging (CMR) examinations for comparison of CMR variables.

**Table 1:** Characteristics of PCS patients and controls with Echo and ECG examination.

	CON (Echo)	PCS
Demographics	*N* = 20	*N* = 100
Age (years)	14.4 ± 2.7	13.2 ± 2.9
Sex, Female, *n* (%)	9 (60%)	56 (56%)
Height (cm)	157.8 ± 37.8	161.2 ± 15.8
Weight (kg)	60.0 ± 16.4	52.9 ± 16.2
BSA (m^2^)	1.7 ± 0.3	1.5 ± 0.3
6-MWD (m)	–	444.0 ± 82.9
Laboratory values		
Troponin T (ng/L)	–	2.3 ± 0.3
NT-proBNP (ng/L)	–	27.1 ± 4.2
CRP (mg/L)	–	1.0 ± 0.2
IL-6 (ng/L)	–	2.3 ± 0.1
ESR 1 h (mm)	–	7.1 ± 0.6
ESR 2 h (mm)	–	15.3 ± 1.3
Symptoms		
Reduced exercise capacity	–	90%
Lack of concentration	–	65%
Dyspnea	–	49%
Headache	–	49%
Fatigue	–	41%
Sleeping disorders	–	39%
Muscle pain	–	34%
Vertigo	–	28%
Stomach pain	–	23%
Chest pain	–	23%
ECHO Variables		
LV-EF Bullet (%)	63.1 ± 1.2	64.0 ± 0.6
MV E/A Ratio	1.9 ± 0.1	2.3 ± 0.3
AoV VTI (cm^3^)	26.1 ± 0.9	26.9 ± 0.5
RVAWd (cm)	0.43 ± 0.03	0.38 ± 0.01
RVEDD (cm)	2.0 ± 0.10	1.8 ± 0.05
TAPSE (cm)	2.4 ± 0.07	2.3 ± 0.04
TV E/A Ratio	1.7 ± 0.08	1.8 ± 0.05
LV GLS (%)	−27.4 ± 0.6	−27.4 ± 0.3
RV GLS (%)	−25.1 ± 0.8	−26.9 ± 0.4
ECG Variables		
HR (bpm)	69 ± 2.7	74 ± 1.5
P-width (ms)	70.0 ± 2.1	86.9 ± 1.5
PQ-time (ms)	134.8 ± 7.5	137.3 ± 2.2
QRS-time (ms)	89.5 ± 2.8	87.9 ± 1.0
QTc (ms)	411.7 ± 5.5	422.5 ± 2.2

Demopgraphic data of PCS patients (Echo cohort) and age-matched healthy controls. Laboratory studies included serum NTproBNP and Troponin c. Data are are mean ± SD for demographic data, and mean ± SEM for laboratory, Echo and ECG values. Symmetrically-distributed data were compared using the unpaired *t*-test, while the Mann–Whitney test was performed on skewed data.

AoV, aortic valve; BNP, brain natriuretic peptide; BSA, body surface area; CRP, c-reactive protein; ESR, erythrocyte sedimentation reat; IL−6, interleukin 6; LV, left ventricle; LV EF, left ventricular ejection fraction; LVGLS, left ventricle global longitudinal strain; MV, mitral valve; 6-MWD, six-minute walk distance; PCS, Post-COVID syndrome; RV, right ventricle; RVAWd, right ventricular wall thickness; RVEDD, right ventricular end-diastolic diameter; RVGLS, right ventricular global longitudinal strain; TAPSE, tricuspid annular plane systolic excursion; TV, tricuspid valve.

Patients had persistent PCS-typical symptoms 12 weeks after PCR-confirmed SARS-CoV-2 infection. The most common symptoms were reduced exercise capacity (90%), lack of concentration (65%), dyspnea (49%), headache (49%), fatigue (41%), sleeping disorders (39%), Muscle Pain (34%) and Vertigo (28%). All 100 pediatric PCS patients underwent blood laboratory examination with determination of Troponin T, NT-proBNP, inflammation markers (CRP, IL-6, ESR 1 h and 2 h), ECG and detailed Echo examination (Table 1). A control cohort of 20 age-matched healthy children served as a comparison group, who underwent ECG and Echo examination from the same pediatric cardiologist and protocol.

42 of the 100 PCS patients, in which no sedation was necessary, additionally underwent a comprehensive CMR examination in which conventional CMR variables as well as right and left ventricular strain were determined, in order to sensitively assess myocardial contractility. CMR examinations in PCS patients were perfomed 2 days up to a maximum of 8 weeks after initial Echo examination. A control cohort of 28 age-matched healthy children received identical protocol-driven CMR examinations and served as comparison group for CMR variables.

### 2.2 Transthoracic Echo, including 2D-speckle tracking

Biventricular function, wall-/chamber-diameters and flow rates were assessed using echocardiographic B-mode, M-Mode, PW- and CW-Doppler, and ventricular strain analysis. Transthoracic echocardiography was performed on two ultrasound machines: a Philips iE33 ultrasound system and a Philips EPIQ 7 ultrasound system, with 5–1, 8–3, and 12–4 MHz transducers, depending on age, height and weight of the patient. Images were recorded digitally and analyzed using the IntelliSpace Echo software (Philips Medical Systems, Netherlands, Veenplius) by a single experienced investigator. To sensitively judge myocardial contractility we assessed additional Echo strain analysis using TomTec 2D-Autostrain software (Version 2.41.00, TomTec Imaging Systems, Unterschleissheim, Germany).

### 2.3 ECG

All patients and controls received a conventional 12-lead ECG. The conventional ECG times and the QRS angle were determined in lead II-Einthoven.

### 2.4 CMR, including CMR strain analysis

CMR imaging was performed on a 3.0 T MRI Scanner (Magnetom, Siemens Healthcare, Erlangen, Germany) in non-sedated, awake patients and controls. The offline analysis was carried out with the software cvi42 (Version 5.11.1, Circle Cardiovascular Imaging, Calgary, Canada) and included conventional CMR variables such as left- and right ventricular ejection fraction, volumes and mass as well as detailed strain analysis with determination of longitudinal, circumferential and radial right and left ventricular strain.

### 2.5 Statistical analysis

Statistical analysis were performed with the Software GraphPad Prism (Version 6.0, GraphPad Software, Boston, USA). First, the values of the PCS and control group to be compared were tested for gaussian normal distribution. Symmetrically-distributed data were compered using the unpaired *t*-test, while the Mann–Whitney test was performed on skewed data. *P* values < 0.05 were considered significant.

## 4 Results

**Figure 1:**
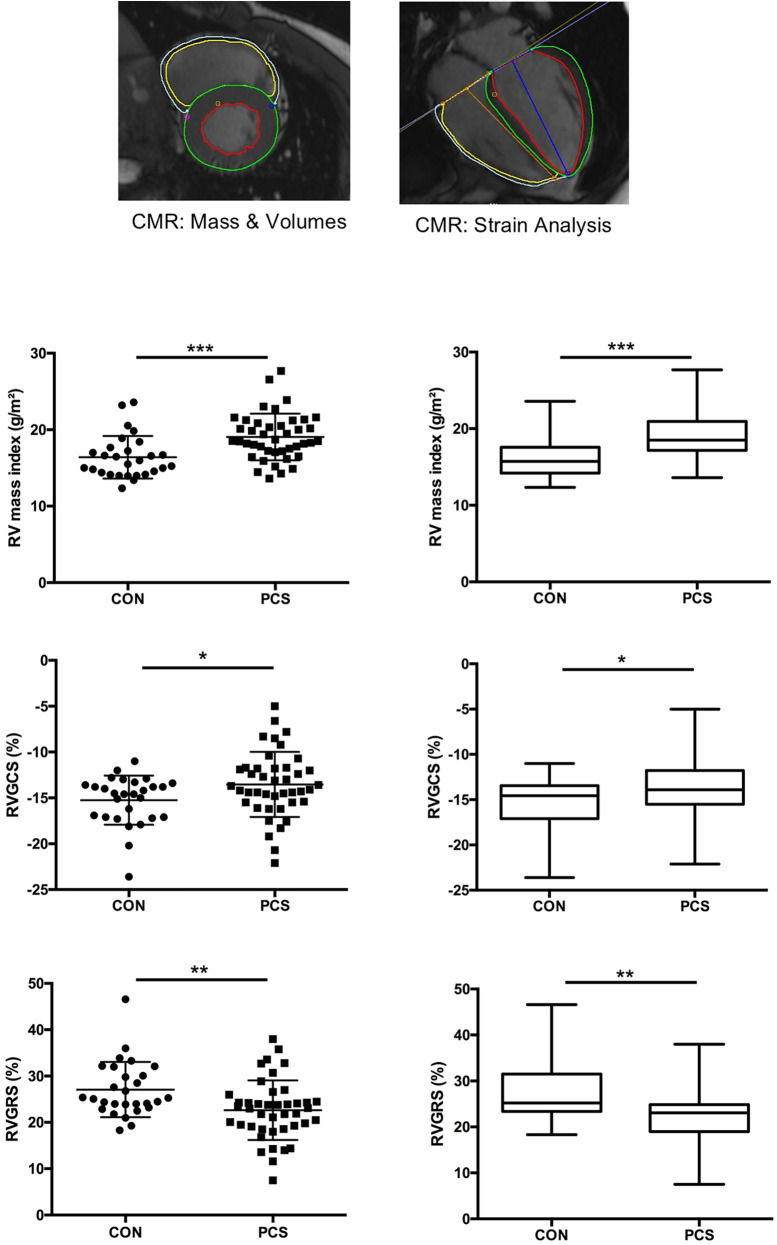
CMR-derived phenotyping ndemonstrated moderately decreased RV contractility and increased RV mass in children and adolescents with post-COVID syndrome (PCS). 42 out of 100 pediatric PCS patients as well as 28 age-matched controls (CON) underwent comprehensive cardiac magnetic resonance (CMR) examination for the determination of conventional CMR variables (ventricular mass and volumes, ejection fraction) and CMR-derived biventricular strain variables (3.0 T MRI system Magnetom Vida, Siemens Healthineers, Erlangen, Germany). The scatter plots on the left and the whiskers plots on the right provide data for RV mass index (upper panel), RV global circumferential Strain (RVGCS; middle panel), and RV global radial Strain (RVGCS; lower panel). The scatter plots show the mean ± SEM; the box and whiskers plots show the median with IQR ± 10–90th centile. *p < 0.05, **p < 0.01, ***p < 0.001.

Complete and detailed results as well as the most common symptoms in pediatric PCS are shown in Table 1 (Echo, ECG) and Table 2 (conventional CMR, CMR Strain). Significant differences (*p* value < 0.05) in CMR parameters are shown in Figure 1. Only 9 of 100 pediatric PCS patients had a slightly elevated NTproBNP between 100 and 200 ng/L, the remaining patients had an NTproBNP <100 ng/L. Inflammation markers (CRP, IL-6, ESR) were not elevated in PCS patients. None of the PCS patients showed a pathologically elevated troponin T value. We saw no significant difference (*p* value > 0.05) in the conventional echocardiographic parameters between the PCS patients and the control group. In particular, left ventricular ejection fraction (LVEF), right ventricular (RV) dimensions (RVEDD, RVAWd) and tricuspid annular plane systolic excursion (TAPSE) did not differ significantly. RV longitudinal global and free-wall strain derived by Echo showed normal values in both groups. The ECG times as well as basal heart rates did not differ significantly between the two groups. In both groups our results showed age-appropriate normal values for ECG times (PQ, QRS, QTc) and heart rate at rest.

**Table 2:** Characteristics of PCS patients and controls with CMR examination.

	CON	PCS
Demographics	*N* = 28	*N* = 42
Age (years)	14.2 ± 3.1	14.3 ± 2.5
Sex, Female, *n* (%)	14 (50%)	26 (62%)
Height (cm)	163.5 ± 14.7	164 ± 12.6
Weight (kg)	56.9 ± 18.9	54.7 ± 16.5
BSA (m^2^)	1.60 ± 0.1	1.52 ± 0.27
6-MWD (m)	–	451.31 ± 76.8
Laboratory values		
Troponin T (ng/L)	–	1.8 ± 0.3
NT-proBNP (ng/L)	–	20.4 ± 4.9
CRP (mg/L)	–	1.0 ± 0.3
IL-6 (ng/L)	–	2.2 ± 0.1
ESR 1 h (mm)	–	5.8 ± 0.8
ESR 2 h (mm)	–	12.9 ± 1.7
Symptoms		
Reduced exercise capacity	–	95,2%
Dyspnea	–	71,4%
Lack of concentration	–	64,9%
Sleep disorders/Insomnia	–	38,1%
Muscle pain	–	38,1%
Headache	–	35,7%
Fatigue	–	35,7%
Vertigo	–	33,3%
Chest pain	–	33,3%
Stomach pain	–	23,8%
CMR Variables		
LVEDV index (ml/m^2^)	76.6 ± 2.3	79.1 ± 2.2
LV-EF (%)	64.8 ± 0.6	65.2 ± 0.8
LV mass index (g/m^2^)	52.3 ± 1.6	54.2 ± 1.7
RVEDV index (ml/m^2^)	90.7 ± 3.0	92.1 ± 2.7
RV-EF (%)	53.5 ± 0.6	50.9 ± 1.0
RV mass index (g/m^2^)	16.4 ± 0.5	19.1 ± 0.5
RVGLS (%)	−24.6 ± 0.6	−24.5 ± 0.6
RVGCS (%)	−15.3 ± 0.5	−13.0 ± 0.8
RVGRS (%)	27.1 ± 1.13	22.6 ± 1.0

Demopgraphic data of PCS patients (Echo cohort) and age-matched healthy controls. Laboratory studies included serum NTproBNP and Troponin c. Data are are mean ± SD for demographic data, and mean ± SEM for laboratory and CMR values. Symmetrically-distributed data were compared using the unpaired t-test, while the Mann–Whitney test was performed on skewed data.

BNP, B-type natriuretic peptide; BSA, body surface area; CON, controls; LV, left ventricle; CRP, c-reactive protein; ESR, erythrocyte sedimentation reat; IL-6, interleukin 6; LVEF, left ventricular ejection fraction; PCS, post-COVID syndrome; RV, right ventricle; RVEDV, right ventricular end-diastolic volume; RVEF, right ventricular ejection fraction; RVGLS, right ventricular global longitudinal strain; RVGCS, right ventricular global circumferential strain; RVGRS, right ventricular global radial strain.

### 4.1 CMR strain parameters and RV mass index are significantly altered in PCS

CMR-based RV mass index was increased in PCS compared to CON (19.06 ± 0.47 vs. 16.4 ± 0.53 g/m^2^; *p* = 0.0002). CMR-derived RV global radial strain (RVGRS; 22.6 ± 1.0 vs. 27.1 ± 1.13%; *p* = 0.003) and RV global circumferential strain (RVGCS; −13.5 ± 0.55 vs. −15.2 ± 0.51%; *p* = 0.045) were significantly decreased in PCS vs. CON. Children with PCS also tended to have mildly reduced RVEF (50.9 ± 0.80 vs. 53.5 ± 0.66%; *p* = 0.259) (Figure 1), though in normal range referred to age-appropriate normal values. In contrast, CMR-derived LV variables (LVEF, LVEDV, LVESV, LV mass), including tissue tracking (strain) analysis (LVGLS, LVGCS, LVGRS), revealed similar values in PCS and control subjects. In both groups, there were outliers in the higher and lower ranges**.**

## 5 Discussion

Worldwide, about 6 million people have experienced PCS so far, which makes PCS a relevant public health issue (8). 25% of 80,071 children with SARS-CoV-2 infection were affected by LC, with the most frequently reported clinical complaints being mood symptoms (16.50%), fatigue (9.66%), and sleep disorders (8.42%) (9).

PCS is nowadays considered a multisystemic condition of persistent symptoms following resolution of an acute severe acute SARS-CoV-2 infection. While this syndrome spans multiple organ systems, cardiovascular complications are often the most prominent features in adults, and include, but are not limited to, myocardial injury, heart failure, arrhythmias, vascular injury/thrombosis and dysautonomia (2, 10). Adult PCS patients have systemic inflammation and immune dysregulation compared to fully recovered Covid-patients. In particular, CD4+ and CD8+ positive *T*-cell numbers and SARS-CoV-2 antibody concentrations are increased in the peripheral blood of PCS vs. non-PCS patients, and there is mis-coordination between their SARS-CoV-2-specific T- and B-cell responses (11). In a large, prospective Long-Covid study, adult PCS patients (*N* = 346) showed mild functional impairment in several CMR variables (RVEF, LVEF). RVEF was significantly lower in PCS vs. 95 control subjects (54.0 ± 5.6 vs. 56.0 ± 5.8; *p* = 0.002), but only 1.4% had truly abnormal RVEF values, which is consistent with our pediatric study. The majority of adult PCS patients showed persistent cardiac inflammation in T-1 and -2 Mapping but normal serum hs-troponin T concentrations (12). Diffuse myocardial edema was more pronounced in participants who remained symptomatic at follow-up as compared to those who improved (12). However, it should be taken into account that even mild impairment in myocardial integrity and cardiac function over longer periods of time could have cumulative effects, decreasing physical fitness, that affect quality of life and long-term somatic and mental health. A recent meta-analysis of PCS in children and adolescents with symptoms for 4–12 weeks or ≥12 weeks (*n* = 80,071; 21 studies), reported the prevalence of PCS (LC) was 25.24%, and the most prevalent clinical manifestations were mood symptoms (16.50%), fatigue (9.66%), and sleep disorders (8.42%) (9). Our study is somewhat limited as we performed deep standardized cardiovascular phenotyping in consecutive children with PCS at the time of diagnosis, but not at a later timepoint. The long-term consequences of the moderate impairment of RV contractility and altered RV morphology (increased RV mass) after SARS-CoV-2 infection are not yet fully understood. A possible association between the cardiac dysfunction and specific coronavirus variants or vaccination are unknown. In our study, patients were not differentiated according to virus variant, vaccination status and severity of initial infection, which limits our study regarding a potential risk stratification. The limited number of controls in our study may introduce potential biases and reduce the statistical power. Future studies with larger control cohorts and comprehensive assessment of relevant risk factors should include multivariable modelling using binary logistic regression to improve the statistical power. Taken together, Post-COVID syndrome in children and adolescents is associated with decreased CMR-based radial and circumferential RV contractility (RVGRS, RVGCS) and increased RV mass, compared to healthy, age/gender matched controls. In contrast, LV contractility (strain) and mass were not affected. CMR-based tissue tracking (strain analysis) appears to be more sensitive than echocardiographic strain analysis in detecting abnormal myocardial contractility. Whether the aforementioned RV alterations are causal for the reported cardiopulmonary exercise limitations in pediatric PCS is unknown, and should be investigated in future studies. In view of the current data, it is crucial that children with SARS-CoV-2 infection or PCS undergo comprehensive cardiac diagnostic evaluation and follow-up to identify and treat potential complications at an early stage. Future studies should focus on understanding the mechanisms of altered cardiac function and morphology in order to develop appropriate intervention strategies.

## Data Availability

The original contributions presented in the study are included in the article/Supplementary Material, further inquiries can be directed to the corresponding author.
